# The G Protein-Coupled Receptor Kinases (GRKs) in Chemokine Receptor-Mediated Immune Cell Migration: From Molecular Cues to Physiopathology

**DOI:** 10.3390/cells10010075

**Published:** 2021-01-05

**Authors:** Marta Laganà, Géraldine Schlecht-Louf, Françoise Bachelerie

**Affiliations:** Inflammation Chemokines and Immunopathology, INSERM, UMR 996, Faculté de Médecine, Université Paris-Sud, Université Paris-Saclay, 92140 Clamart, France; marta.lagana@universite-paris-saclay.fr

**Keywords:** G protein-coupled receptor kinases (GRKs), chemokines, G protein-coupled receptors (GPCRs), atypical chemokine receptors (ACKRs), immune cell migration, chemoattractant gradients, signalling, desensitization, scaffolding

## Abstract

Although G protein-coupled receptor kinases (GRKs) have long been known to regulate G protein-coupled receptor (GPCR) desensitization, their more recently characterized functions as scaffolds and signalling adapters underscore that this small family of proteins governs a larger array of physiological functions than originally suspected. This review explores how GRKs contribute to the complex signalling networks involved in the migration of immune cells along chemokine gradients sensed by cell surface GPCRs. We outline emerging evidence indicating that the coordinated docking of several GRKs on an active chemokine receptor determines a specific receptor phosphorylation barcode that will translate into distinct signalling and migration outcomes. The guidance cues for neutrophil migration are emphasized based on several alterations affecting GRKs or GPCRs reported to be involved in pathological conditions.

## 1. Introduction

G protein-coupled receptor kinases (GRKs) encompass seven protein isoforms that belong to three different subfamilies: the visual subfamily including GRK1 and GRK7, the GRK2 family including GRK2-3 and the GRK4 family composed of the GRK4, -5 and -6 isoforms. GRK2-3 and GRK5-6 proteins are ubiquitously expressed in cells and tissues, while GRK1 and GRK7 are localized in the retina, and GRK4 is mainly localized in the testis [1]. These proteins were initially characterized by their essential and specialized role in the phosphorylation and desensitization of seven transmembrane domain (7TM) receptors coupled to G proteins (GPCRs), the largest family of receptors with many implications in human health and disease. Numerous studies have since established that GRKs also engage noncanonical functional and scaffolding interactions with cellular partners and may even act via kinase-independent mechanisms. The mechanisms connecting these dual functions of GRK to control the biological functions of GPCRs are excessively complex and not completely elucidated. Deletion of the GRK2-3 or GRK5-6 isoforms in mice results in numerous disorders in organismal homeostasis, with notable consequences that have been linked to immune dysfunction (i.e., immune deficiency and inflammatory diseases), consistent with the high expression levels of these GRKs in immune cells. Although the molecular mechanisms underlying the contribution of GRK expression to immune homeostasis and responses are not completely understood, a large body of evidence reveals a link between these GRK2-3 and GRK5-6 isoforms and the chemokine receptor subfamily of GPCRs (Table 1). The present review will focus primarily on these interactions and summarize genetic and pathophysiological evidence that have provided insights into the role of GRKs in immune cell homeostasis. Specifically, we will first discuss studies that refer to the balanced roles of GRKs in GPCR desensitization and activation and their regulation that underlies their actions on chemokine receptors. Then, we will highlight recent advances in understanding how the GRK-dependent regulation of chemokine receptor activation is rewired by modulating the conformation of the receptor, which is notably induced by the binding of intracellular effectors or the nature of the chemokine ligand, by emphasizing some receptors (e.g., CXCR4, CCR7 and CXCR1/2). We will conclude by discussing the current knowledge of how these molecular cues and the regulation of GRK expression translate into myeloid immune cell trafficking in physiological and pathological contexts.

## 2. Linking GRK Deficiencies to Immune Dysfunction

Several genetic mouse models have provided clues to the importance of GRKs in physiology, as extensively discussed elsewhere [16]. Concerning the ubiquitously expressed GRK2/3 subfamily, a loss of GRK2 mostly induces cardiac developmental defects causing embryonic lethality [17], while GRK3 knockout mice display impaired olfaction and neuronal functions but are viable [18], thus suggesting different targets and physiological functions for the members of this subfamily. The links between GRKs disruption and immune dysfunction came from deeper studies in knockout mice, including models with tissue-specific deletion, and prompted investigations of their molecular bases. Pathophysiological manifestations of mice harbouring a macrophage-specific deficiency of GRK2 pinpoint a role for this kinase in myeloid cell homeostasis [19] and in limiting their activation in inflammatory conditions [20]. Conversely, sepsis-associated inflammation is reduced in GRK5-deficient mice [21], suggesting opposite effects of GRK2 and GRK5 on myeloid cells and/or effects in other cell types. Several immune impairments are also reported in GRK3-deficient mice, including an increase in the number of haematopoietic stem cells (HSCs) biased towards myeloid/granulocyte subsets in the bone marrow (BM) [12]. Interestingly, mice lacking GRK6 [22,23] exhibit lymphopenia [24], although GRK6 does not appear to be required for haematopoiesis [25,26]. The anomalies reported in GRK3- and GRK6-deficient mice are reminiscent of the features characterizing the rare WHIM immunodeficiency syndrome [27] reported in the early 1960s. The acronym for the WHIM syndrome is based on the clinical manifestations of human papillomavirus virus (HPV)-induced Warts, Hypogammaglobulinemia, recurrent bacterial Infections and pathognomonic Myelokathexis, which refers to an abnormal increase in the number of senescent neutrophils in the BM [28,29]. Marked panleukopenia completes the picture [30], as phenocopied by a mouse model of the syndrome [31]. Considering that the WHIM syndrome is caused by gain of function mutations in *CXCR4* gene, these observations support the critical role of GRKs in controlling this chemokine receptor’s functions.

From the molecular perspective, GRKs are thought to primarily control immune processes by regulating cell migration in both homeostatic and inflammatory conditions through their action on chemokine receptors that are master regulators of immune cell trafficking. In humans, 18 of 23 chemokine receptors are GPCRs that trigger canonical pathways upon agonist activation, leading to GRK-mediated phosphorylation of the receptor intracellular domain and subsequent binding of arrestins, a physiological feedback mechanism of homologous desensitization that rapidly uncouples the receptor from G proteins [32,33]. While the enhanced chemotaxis of leukocytes and neutrophils derived from GRK3- [12] and GRK6-deficient mice [26], respectively, toward CXCL12 are consistent with such a classical role for GRK3 and GRK6, the reduced migratory responses of GRK6-/- lymphocytes reveal unexpected positive regulatory effects of GRK6 [22]. The multifunctionality of GRKs and the mechanisms regulating their versatility, including their interacting partners and their expression levels, are becoming better understood [1,34] and are described below.

## 3. Roles of GRKs in Regulating Chemokine Receptor Activation

### 3.1. More Than Governing Desensitization

The chemokine system encompasses chemokine ligands, and their 7TM receptors (~45 and 23, respectively, identified to date) generally display redundancy and binding promiscuity, with the notable exception of CXCL12 and its typical and atypical receptors, CXCR4 and ACKR3, respectively [35]. These receptors are highly conserved across vertebrate phylogeny in accordance with their critical homeostatic functions. As for other GPCRs, chemokine receptors contain an intrinsically disordered intracellular C-ter, whose conformational flexibility contributes to the recruitment of multiple effectors with an underappreciated role in biological outcomes [36]. A paradigmatic function of chemokine receptors is the coordination of cell polarization and directional leukocyte migration towards their chemokine ligands [37,38]. Much remains to be elucidated regarding this multistep process that relies on the tight spatial and temporal control of G proteins and arrestin (i.e., β-arrestin 1 and 2)-dependent signalling pathways transiently set in motion downstream the chemokine-activated receptors. These processes underpinned by the phosphorylation of activated receptor and their desensitization mediated by arrestins orchestrate cycles of adhesion and polarization/elongation, ensuring cell migration towards a chemoattractant. Accordingly, arrestins have emerged as important regulators of cell migration both via their essential role in receptor desensitization and endocytosis (the link between endocytosis and cell migration is reviewed elsewhere [39]) and via their signalling activity; the molecular mechanisms underlying these processes and their relative contributions to migration control are still a matter of intense research [40,41].

A large body of literature has established the critical importance of GRKs in initiating the process of desensitization of chemokine receptors in a kinase-dependent manner via arrestin recruitment, as summarized in Table 1. This role of GRKs manifests by increased chemotaxis toward chemokine gradients upon GRK invalidation. For examples, the observation that neutrophils from GRK6-deficient mice were more responsive to CXCL12 than their wild-type (WT) counterparts was interpreted as reflecting a role for GRK6 in the CXCL12-induced desensitization of CXCR4 [26], which was as also proposed for GRK3 [12]. In contrast, the CXCR4-dependent chemotaxis of lymphocytes towards CXCL12 was impaired in GRK6-deficient mice as in β-arrestin 2-deficient mice [22], providing the first in vivo proof-of-concept for a role of these two regulators of GPCR desensitization in promoting cell migration.

**Figure 1:**
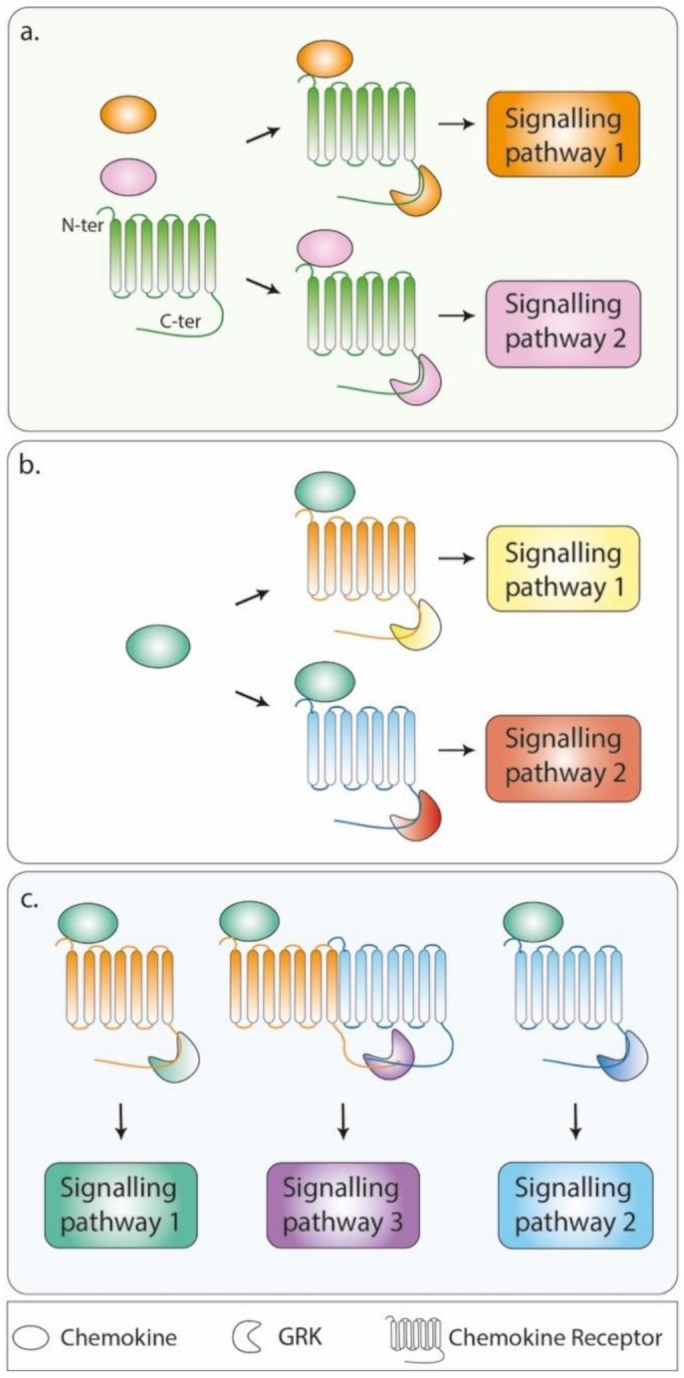
Chemokines and their receptors bias the G protein-coupled receptor kinase (GRK)-associated phosphorylation barcode. According to this bias, receptors adopt different conformational states that mask or unmask specific serine or threonine sites for GRK-dependent phosphorylation. (**a**) Different ligands may bind the same chemokine receptor, promoting the differential recruitment of GRKs that activate distinct signalling pathways. An example of this situation is illustrated by the trio CCL19/CCL21-CCR7 (green receptor), whereby, in dendritic cells (DCs), CCL19 (orange oval) is promoting haptotaxis in a GRK3-dependant manner (orange form) (Signalling pathway 1), while CCL21 (pink oval) is promoting chemotaxis in a GRK6-dependant manner (pink form) (Signalling pathway 2). (**b**) A single chemokine may bind to several receptors, differentially recruiting GRKs that activate distinct downstream pathways. For instance, CXCL8 chemokine (green oval) binding to CXCR2 (orange form) is promoting the GRK6-dependant phosphorylation and activation of receptor downstream signalling pathways in the initial phase of cell migration (Signalling pathway 1). Its binding to CXCR1 (blue form) is promoting cell chemotaxis and GRK2-dependent phosphorylation and endocytosis of the receptor (Signalling pathway 2). (**c**) Chemokine receptors have the propensity to form oligomers to which the binding of the chemokine is anticipated to result in the activation of different signalling pathways (Signalling pathway 3) than the ones resulting from the engagement of each receptor. For sake of simplicity, dimers are depicted.

Taken together, these results prompted several hypotheses with regard to the regulatory role of GRKs in chemotaxis. Different GRKs might be recruited to the receptor upon chemokine binding and interact with distinct domains of the receptor, according to the availability of the other components of the signalling pathways that are activated (e.g., heterotrimeric G proteins, small G proteins and their exchange factors, extracellular receptor kinase mitogen-activated protein kinases (ERK1/2 and MAPKs)) (Figure 1a). This coordinated action of different GRKs would be translated into distinct biological outcomes upon phosphorylation barcoding [42,43,44], which is envisioned as a general signalling hub that would be biased by the conformation of the receptor notably induced by the binding of arrestins and cellular effectors, the nature of the chemokine ligand (Figure 1b) and the possible allosteric interactions with other receptors (i.e., oligomerization, Figure 1c), including atypical chemokine receptors, which are known for their regulatory effect on chemoattractant signals. Functional evidence for this concept of phosphorylation barcoding was provided by recent studies revealing that switching the code pattern of phosphosites between GPCRs reprograms arrestin activation [45,46].

### 3.2. Patterning the Phosphorylation Code: Focus on CXCR4

The phosphorylation barcoding hypothesis proposes that each GRK preferentially phosphorylates a limited number of distinct serine and threonine (Ser and Thr) residues on a particular GPCR, thus creating a barcode. While other protein kinases (e.g., PKC) mediate agonist-dependent phosphorylation of activated GPCRs, their roles are beyond the scope of this review [14,47,48]. The conformations of the arrestins recruited to the phosphorylated residues and their scaffolding actions would further dictate specific signalling outcomes.

**Figure 2:**
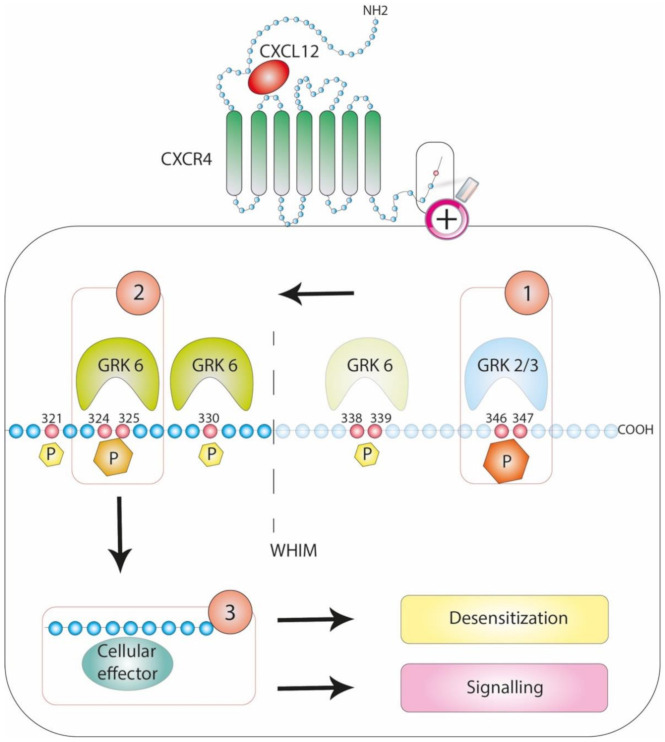
Hierarchical phosphorylation pattern at the C-ter of CXCR4. Upon CXCL12 binding to CXCR4, a cascade of phosphorylation takes place at the C-ter of the receptor following a hierarchical order, as depicted by the numbering. Subsequently, cellular effectors (e.g., arrestins and the COMMD3/8 complex) are recruited and promote signalling and receptor desensitization. The absence of the dominant 346/347 phosphosites in Warts, Hypogammaglobulinemia, recurrent bacterial Infections and pathognomonic Myelokathexis (WHIM)-associated CXCR4 C-ter truncation prevents CXCR4 desensitization. The most common C-ter truncation occurring in WHIM patients is depicted by a dashed line and faded residues.

Several reports support the possibility of these mechanisms for CXCR4, the broadly expressed G protein (mostly the inhibitory Gi family member)-coupled receptor of the CXCL12 chemokine. This pair, which exhibits a high degree of conservation throughout evolution from jawless fish to humans, is essential for embryogenesis, including proper haematopoiesis, and plays nonredundant functions in immune homeostasis [49,50]. An overview of the current knowledge of CXCR4 interactomes along with their regulation and pathophysiological functions was recently published [51]. Early studies that have identified the role of CXCL12/CXCR4 in human immunodeficiency virus (HIV) pathogenesis [52,53], later in cancer progression and metastasis [54] and more recently in the pathogenesis of WHIM syndrome [55] have facilitated research devoted to the regulation of this signalling axis. CXCR4 is rapidly phosphorylated and internalized upon CXCL12 engagement in a manner dependent upon its intracytoplasmic C-terminal domain (C-ter) [56,57]. Alanine scanning mutagenesis has suggested that the 15 Ser and 3 Thr residues encompassing the 45-residue-long CXCR4 C-ter may be phosphorylated [58]. A more accurate identification of the sites potentially phosphorylated upon CXCL12 stimulation was performed in the human embryonic kidney 293 cell line [6]. Busillo et al. also proposed that GRK6- and GRK3-, as well as GRK2-mediated phosphorylation, targets different residues with distinct effects on arrestin recruitment, conformation and activation, thus determining signalling outcomes (Figure 2). Indeed, although the recruitment of β-arrestin 2 to GRK6- and GRK3-induced phosphosites would result in CXCR4 desensitization, the recruitment of β-arrestin 1 would favour ERK1/2 activation [6]. More recently, phosphoselective CXCR4 antibodies were instrumental in providing supporting evidence for the hierarchical use of the phosphosites contained in the CXCR4 C-ter, suggesting that a single site might dictate whether other sites become efficiently phosphorylated [7].

The disruption of the phosphorylation barcode through mutations in the C-ter of a chemokine receptor may have serious pathological consequences, as illustrated by WHIM syndrome. This syndrome is the first example of a human disorder mediated by dysfunctions of a chemokine receptor, namely CXCR4, and is mostly linked to inherited heterozygous autosomal dominant mutations in the CXCR4 gene [55]. All eleven mutations described among the 105 patients reported to date target the domain encoding the receptor C-ter, mostly premature stop codons that eliminate the last 10 to 19 C-ter residues, some frameshifts that introduce 3 to 24 additional new amino acids and one missense mutation [59,60]. Impaired CXCR4 internalization upon CXCL12 stimulation is a hallmark of patient-derived cells and cell lines expressing WHIM-associated mutants [61,62], despite the conservation of some phosphosites (e.g., serine residues Ser324/Ser325). This phenomenon might be attributed to the proposed hierarchical organization of CXCR4 C-ter phosphosites, whereby CXCL12-induced phosphorylation at Ser324/Ser325 residues depends on the phosphorylation of more distal residues (i.e., Ser346/Ser347 residues) that are deleted in WHIM-associated mutants [7] (Figure 2). Along this line, Nakai et al. recently provided a model of this type of modulatory process whereby CXCL12-induced murine B cell chemotaxis is associated with the phosphorylation of the CXCR4 C-ter by GRK2/3, and then, the recruitment of the COMMD3/8 complex, a potential susceptibility factor for inflammation [63], promotes the recruitment of GRK6 that will subsequently phosphorylate far CXCR4 C-ter residues and promote β-arrestin 2 binding [8]. Similar findings were reported for the β2-adrenergic receptor [8], which is also involved in inflammation [64], supporting the general interplay between GRK3, GRK6 and arrestin-mediated signalling.

This organization would provide an explanation of why and how the different WHIM-associated mutations translate into broad dysfunction downstream of the CXCL12/CXCR4 axis. Along with this phosphorylation pattern, investigations of the pathogenesis of WHIM syndrome permitted us to assign a major role to GRK3 in regulating CXCL12-induced desensitization of CXCR4. Indeed, on the one hand, the impairment of CXCL12-induced CXCR4 internalization harboured by cells derived from patients with WHIM lacking CXCR4 mutations [61] was attributed to selective alterations in GRK3 activity [13]. On the other hand, the pattern of CXCR4 phosphorylation analysed in cell lines using tandem mass spectrometry and phosphosite-specific CXCR4 antibodies identified the importance of GRK2/3 in the CXCL12-induced phosphorylation of the dominant Ser346/Ser347 residues [7,14]. Finally, leukocytes derived from GRK3-deficient mice display impaired CXCL12-induced CXCR4 endocytosis, abnormally enhanced chemotaxis and prolonged ERK1/2 activation [12].

### 3.3. Driving Signalling Pathways

The enhanced and prolonged CXCL12 responses featured in WHIM syndrome (i.e., ERK1/2 signalling and chemotaxis) may also result from aberrant activation of arrestin-dependent pathways, as originally revealed by the impaired migration of leukocytes from β-arrestin 2 knockout mice in response to CXCL12 [22] and further supported by the seemingly paradoxical strengthened CXCL12-induced chemotaxis by β-arrestin 2-dependent signalling in human cells [65,66]. Indeed, WHIM-associated C-ter-deleted CXCR4 mutants were unexpectedly shown to maintain an association with β-arrestin 2 [67,68] that relies on the third intracellular loop (ICL3) that binds the actin-binding protein filamin A [69] and would contribute to the increase CXCL12-induced cell chemotaxis. Recent cryoelectron microscopy-based structures of β-arrestin 1 in complex with two GPCRs clarified the general contribution of the ICL3 domain to the receptor interaction with arrestins [70,71]. This interaction between the ICL3 domain of the WHIM-associated CXCR4 mutant and arrestin is central in triggering increased arrestin-dependent signalling (i.e., ERK1/2 signalling) upon CXCL12 stimulation [67]. This process, together with the impaired desensitization of CXCR4, causes the gain of CXCL12/CXCR4 function that characterizes WHIM syndrome. Thus, this pathological condition provides proof of principle of the contribution of both signalling and internalization functions of arrestins in chemotaxis [41]. It also supports the hypothesis that the receptor conformations induced by the differential binding of arrestins or other components of the signalling pathways could bias the phosphocode (i.e., the specificity of GRK targeting and recruitment) and, ultimately, the biological and potential pathological outcomes. In this respect, further investigations are needed to determine whether these mechanisms also guide the pathogenesis of Waldenstrom’s macroglobulinemia, a plasma cell cancer associated with somatic mutations of CXCR4 that are similar to WHIM-associated nonsense mutations and associated with a poorer prognosis [72].

Finally, in light of the multiple protein partners reported to physically interact with GRKs or to display concurrent regulation of their expression with GRKs (reviewed in [1,34,73]), the contribution of GRKs to the control of chemokine receptor activity is suspected to be much broader than recruiting and controlling arrestin functions. Such scaffold activity could give additional regulatory roles to these multidomain proteins, which may even be independent of their kinase function and could rely, in part, on the presence of a pleckstrin homology domain in GRK2/3. With regards to chemokine receptor regulation and, more broadly, immune cells migration, the characterization and contribution of the GRKs interactome is still in its infancy (Table 2) [74]. The main achievements in the field are related to GRK2, which was notably proposed to regulate ERK1/2 signalling pathways by directly interacting with ERK2, MEK (Mitogen-activated protein kinase kinase), Raf kinase inhibitor protein (RKIP) or the small RhoA GTPase protein upon chemokine receptors or epidermal growth factor engagement [75,76,77]. Moreover, GRK2 can negatively regulate ERK activation in response to lipopolysaccharide (LPS) in mouse peritoneal macrophages by binding to the p105 subunit of NF-κB (Nuclear Factor-kappa B) [20] or, conversely, can activate ERK signalling pathways downstream the sphingosine-1-phosphate GPCR (S1P) in epithelial cells by recruiting ADP ribosylation factor (ARF)-specific GTPase-activating proteins (GIT) [78]. Whether scaffold activity underlies the positive effect of GRK6 on CXCL12-induced chemotaxis of lymphocytes [22] remains to be demonstrated, but one may envision that this kinase could potentially interact with effectors other than arrestins [6], thus biasing the receptor conformation and signalling pathways.

### 3.4. Regulating Atypically Atypical Chemokine Receptors

The second receptor for CXCL12 is a member of the smallest subgroup of atypical chemokine receptors, 4, in humans; these receptors do not signal through G proteins and, rather, regulate GPCR-driven chemotaxis by tuning chemokine concentrations in tissues (e.g., by scavenging, transporting and trans-presenting chemokines) [96]. Accordingly, original evidence was provided for ACKR3-expressing cells shaping extracellular CXCL12 levels during embryogenesis, supporting a scavenging role for ACKR3 towards its two chemokine ligands, CXCL11 and CXCL12 [97,98]. This scavenging function maintained and controlled the responsiveness of CXCR4-expressing cells in a self-generated chemokine gradient [99]. Thus, inhibiting ACKR3 expression in epithelial rear cells of the posterior lateral line primordium of the fish embryo blocks the directional migration of mesenchymal cells, further supporting the hypothesis that differential localization and activity of ACKR3 are responsible for steering the CXCR4-expressing cell population in the right direction [99,100].

Importantly, the scavenging function of ACKR3 has also been presented as a mechanism that is notably involved in the proper positioning of neurons in the developing mouse brain [2,101,102] or in tumour metastasis via the egress of CXCR4-expressing cancer cells from the primary tumour site [103,104]. The less well-known expression and functions of ACKR3 in the immune system have been reviewed elsewhere [105]. In addition, ACKR3 may regulate chemotaxis by biasing CXCL12/CXCR4 signalling towards arrestin-dependent pathways. This process would result from the reported propensity of ACKR3 and CXCR4 to form oligomers when they are co-expressed in heterologous expression systems, in which ACKR3 differentially affects CXCL12/CXCR4-dependent signalling through allosteric communication between receptors [106,107,108,109,110]. However, the current view that ACKR3 is an arrestin-biased receptor [111,112] and that its scavenging function requires arrestin [113] has been recently challenged [2,114]. In contrast, the phosphorylation of ACKR3 C-ter Ser/Thr residues appeared mandatory for receptor-mediated chemokine scavenging [2,115], supporting the requirement for GRK2 and likely GRK5 for CXCL12 scavenging and CXCL12-mediated ACKR3 endocytosis [2]. Similarly, GRK2/3-induced phosphorylation of ACKR4, the atypical receptor that scavenges the CCR7 and CCR9 chemokine ligands CCL19/CCL21 and CCL25, respectively [116,117], only partially impedes arrestin recruitment [9] and is otherwise dispensable for the ACKR4 scavenging function [9,116]. These studies question the prevailing concept that agonist-induced GPCR endocytosis recruits arrestins to phosphorylated receptors and raises additional questions related to the nature of the effectors recruited downstream of the phosphorylated ACKRs.

### 3.5. Shaping the Cell Migration Mode Promoted by Chemokine Ligands

Among the chemokine receptors, CCR7 provides insights into how different chemokine ligands, namely CCL19 and CCL21, contribute to receptor functions by activating distinct signalling pathways through the generation of selective conformational changes of the receptor associated with a distinct GKR-induced C-ter phosphorylation barcode and, thus, the differential recruitment of effectors (Figure 1a). Both CCL19 and CCL21 chemokines display similar binding affinity for CCR7 and equally activate G protein-dependent signalling pathways, including chemotactic responses [118]. However, while CCL19 promotes the seryl/threonyl phosphorylation of the CCR7 C-ter, CCL21 rarely does [119]. These distinct phosphorylation patterns were associated with differential activation of GRKs, as CCL19 activates both GRK3 and GRK6 and CCL21 activates only GRK6. The functional outcomes include the recruitment and activation of β-arrestin 2 and subsequent ERK1/2 signalling by both chemokines, while only CCL19 promotes CCR7 internalization [11].

The most prominent structural difference between CCL19 and CCL21, which otherwise share the typical tertiary structure of chemokines, is the highly charged C-ter domain of CCL21 that confers this chemokine with the capacity to interact with the cell surface and extracellular matrix-associated glycosaminoglycans (GAGs), while CCL19 is mostly soluble [120,121,122]. More generally, chemokine/GAG interactions are viewed as major determinants shaping adhesive migration along immobilized gradients of chemokines or haptotaxis, whereas chemotaxis is triggered by soluble forms of chemokines [123]. Along with this concept, the critical functions of CCR7 in the trafficking of dendritic cells (DCs) to and inside secondary lymphoid organs [124] relies on the robustness of DC haptotaxis on gradients of CCL21 immobilized within the lymph node combined with soluble CCL21 and CCL19-induced chemotaxis [125]. Consistent with their earlier observations, Schwarz et al. further identified the importance of GRK6-dependent desensitization in CCL21-promoted haptotaxis, while it was dispensable for CCL21-induced chemotaxis [15]. Further evidence for ligand-biased signalling in the control of CCR7-mediated DC migration is the importance of Scr kinase-dependent phosphorylation of CCR7 for the recruitment of SH2 domain proteins such as phosphatase SHP2, whose activation is important for CCL21-mediated migration in the context of some inflammatory cues [126]. Supporting the importance of the regulation of chemoattractant signalling in determining the mode of migration, GRK3-dependent CCR7 desensitization induced by CCL19 was identified as a central mechanism accounting for the requirement of an increase in the absolute CCL19 concentration over time for persistent long-range directional DC migration [10].

These findings were extended to CXCL12/CXCR4-induced persistent directional neutrophil chemotaxis, whereby GRK3-deficient neutrophils remained able to spatially and temporally sense stable CXCL12 gradients [10]. In addition, CXCR4 desensitization is also critical for the control of the precise arrival of the cells at their location [127]. Collectively, these findings support the assumption that GKR-induced receptor desensitization is a strong negative regulatory feedback mechanism for myeloid cell chemokine-driven chemotaxis. This mechanism participates in an adaptation process that is intimately linked to myeloid cell function in sensing and resolving inflammation in which cells can adapt their migratory responses to the temporal evolution of the chemokine concentration, thus ceasing to migrate when the chemotactic signal is stable [10]. This specific mode of migration might explain differences in cell type-specific migration from mice lacking GRK6, where CXCL12-induced chemotaxis is increased for GRK6^-/-^ neutrophils [26] but decreased for GRK6^-/-^ lymphocytes [22]. Regarding the widespread mode of collective cell migration, recent studies have stressed the importance of intercellular adhesion and communication between rear and front cells for the coordination and directionality of cell cluster migration [128,129]. For instance, rotation between cells at the rear and front positions was proposed to occur in groups of lymphocytes migrating along CCL19 or CXCL12 gradients together with receptor desensitization, thus highlighting an additional level of regulation of cell migration (e.g., chemotaxis, haptotaxis and/or chemorepulsion) and chemokine sensing [100,130].

### 3.6. Modulating Receptor-Dependent Routing: Insights from the CXCL8 Chemokine

The CXCL8 chemokine is the ligand of two highly homologous chemokine receptors, CXCR1 and CXCR2 (Figure 1b). CXCR1 also binds CXCL6 and possibly CXCL7, whereas CXCR2 binds promiscuously to all seven members of the CXCL8 subfamily (CXCL1−3 and CXCL5−8) [35]. The current view is that similarly conserved residues located in the extracellular loops of CXCR1 and CXCR2 are important for receptor activation but induce distinct ligand-induced trafficking of these receptors in vitro; CXCR1 is downregulated in response to CXCL8 in contrast to CXCR2, likely due to different recycling potencies [131,132]. While the consequences of such differential receptor routing on immune responses, particularly on the trafficking of neutrophils for which CXCL8 subfamily ligands are potent chemoattractants driving BM egress [133], remain elusive, recent live imaging in zebrafish provided insights into the mechanism by which neutrophils stop and cluster or disperse and leave the site of inflammation upon reaching a chemoattractant source. In this study, Coombs et al. showed distinct trafficking patterns of the zebrafish homologs of CXCR1 and CXCR2 in response to their respective Cxcl8a and Cxcl8b ligands; Cxcr1 was internalized, while Cxcr2 was not, consistent with the higher incidence of serine residue clusters in the Cxcr1 C-ter than in Cxcr2. The authors further established that Cxcr1 downregulation prevents excessive neutrophil clustering, while the sustained residence of Cxcr2 at the plasma membrane prolongs the downstream signalling required for neutrophil dispersal [134]. These results illustrate how the regulation of chemokine receptor trafficking at the cell level potentially orchestrates dynamic cell responses by integrating complex and complementary chemokine-driven signals and support self-resolving immune cell trafficking [134]. Although Cxcr1-dependent processes were proposed to depend on receptor C-ter phosphorylation, this requirement was not absolute for Cxcr2, which also displayed phosphorylation-independent activities [134], supporting the proposed recruitment of GKR6 signalling complexes downstream of CXCL8-activated CXCR2 [135], whereas CXCR1 was suggested to predominantly interact with GRK2 [4]. Studies aiming to determine how these mechanisms occur in the complexity of the whole organism and the relative expression of CXCR2 and CXCR1 to provide insights into how GPCR signals resulting from one or several receptors control leukocyte trafficking are needed. These notions have been recently reviewed in particular, in view of the intravital imaging of the dynamic migration patterns of immune cell subsets in live anaesthetized mice [136].

## 4. GRKs in the Immune Functions of Chemokine Receptors: Focus on Neutrophils

The molecular connections between chemokine receptors and GRKs have prompted the question of how their relative expression is regulated, a question that has been most particularly addressed in relationship with trafficking neutrophils in the context of inflammation. We will outline studies that advanced our understanding of the mechanisms controlling the activities of GRKs and their effects on neutrophil guidance in health and disease, with a focus on CXCR4 functions, in light of WHIM-associated neutropenia and the deregulation of GRK expression in pathological conditions and their interplay with non-GPCRs.

### 4.1. GRKs in Neutrophil Guidance

As mentioned, the GRK2, 3, 5 and 6 isoforms are broadly expressed in leukocytes, although with differential transcriptional expression patterns among cell lineages and cells with different developmental and activation statuses. Most notably, the Immunological Genome Project [137] has provided insights into the particular abundance of these four GRKs in murine neutrophils and their specificity of expression in relation to the neutrophil lifecycle. For instance, while the GRK2 and GRK6 transcripts are approximately equally well-expressed in neutrophils derived from the BM and spleen and in activated neutrophils (i.e., recovered from thioglycolate-induced peritonitis), GRK3 and GRK5 are specifically upregulated in the spleen and in activated neutrophils, respectively [137]. Neutrophils produced in the BM and continuously released into the blood to patrol tissues [138] are endowed with a large spectrum of properties, including unsuspected nonimmune regulatory homeostatic functions, in support of our growing knowledge of neutrophil heterogeneity [139]. Accordingly, committed neutrophil subsets were identified in the BM, including proliferating precursors that are retained in the BM in a CXCR4-dependent manner [140]. Moreover, Hidalgo and col. showed that neutrophils gain distinct phenotypic and functional properties in healthy tissues into which they are guided by tissue-derived signals, such as in the lungs, where CXCR4 signalling is required to pilot neutrophils in CXCL12 niches [141,142]. This finding suggests the existence of similar instructing wires controlled by the other GPCRs expressed on neutrophils to sense, such as CXCR4, chemokines (CXCL8 subfamily through CXCR1 or 2) or N-formyl peptides (through FPR1 and FPR2), complement component 5a (C5a mainly through C5ar1) or leukotriene B4 (LTB4 mainly through LTB4R1/BLT1) [143], which would guide neutrophils to specific reprogramming tissue areas. Therefore, in-depth analyses of GRK expression in tissues that are targeted by neutrophils are expected to provide additional insights into the mechanisms involved in tissue-dependent functional imprinting.

Compelling evidence indicates a critical role for CXCR4 desensitization in the processes controlling the residence of neutrophils in the BM and their release into the blood. These homeostatic processes are controlled by the balanced interplay between two chemokine systems (CXCL12/CXCR4-ACKR3 and CXCL1, 2 and 8/CXCR1 and 2), where CXCR4 expressed by BM neutrophils is desensitized and internalized in response to high local concentrations of CXCL12 within BM microenvironments, while low levels of CXCR2 ligands expressed by endothelial cells recruit neutrophils out of the retentive CXCL12 domains for entry into the blood circulation in a CXCR2-dependent manner [144,145].

Therefore, the neutropenia that characterizes patients with WHIM [28,60] is understood to result from the transdominant gain of function and impaired desensitization of the mutant CXCR4 allele. First, genetic knock-in strains of zebrafish or mice harbouring the equivalent of the WHIM-associated CXCR4 mutant phenocopied neutropenia [31,146]. Neutropenia is reversed upon treatment of the WHIM mouse model with the selective CXCR4 antagonist, AMD3100 [31], which causes acute neutrophilia in wild-type mice [147]. Furthermore, upon chromothripsis, the WHIM-CXCR4 allele deleted from the myeloid lineage of a patient with WHIM was associated with a correction of neutropenia [148]. In addition, clinical studies based on the chronic use of AMD3100 (i.e., market name of plerixafor) show beneficial effects on the mobilization of neutrophils and most subtypes of leukocytes [60], because patients suffer from panleukopenia, including lymphopenia, consistent with important roles for CXCL12/CXCR4 signalling in regulating haematopoiesis and immunity, as observed in patients and a mouse model of WHIM syndrome [149]. Finally, the still-debated questions of the sources of neutrophils mobilized by AMD3100 together with neutrophil heterogeneity (spatial, phenotypic and functional) have prompted doubts about neutrophil sequestration in the BM as the sole mechanism to explain neutropenia driven by the WHIM-associated gain of CXCR4 function. Other processes might be involved, as also suggested for myelokathexis, which is considered pathognomonic in patients with WHIM, since it has been identified only once elsewhere in two inherited cases of loss-of-function CXCR2 mutations [150] and might also be accounted for by accelerated senescence or clearance of neutrophils.

Neutrophils are short-lived cells in the bloodstream that are cleared from blood and eliminated upon infiltration into the BM, liver and spleen [151], as well as in tissues [142], indicating that several organs are responsible for natural clearance. During this process of ageing, neutrophils undergo phenotypic changes, including a progressive increase in cell surface CXCR4 expression following circadian patterns that engage antagonistic CXCR2- and CXCR4-dependent signalling [152,153]. Bacterial infections and inflammation are associated with neutrophilia in patients with WHIM, similar to healthy individuals, providing a possible explanation for why patients with WHIM do not suffer from invasive life-threatening bacterial infections compared to patients with other types of congenital or acquired neutropenia and survive into adulthood [60]. Nevertheless, the trafficking of neutrophils through inflamed or infected sites, the local execution and the termination of their specific effector functions that will determine the fate between pathogen and inflammation clearance and tissue damage remain challenging questions that have never explored in the context of WHIM-associated CXCR4 gain of function.

### 4.2. Modulation of GRK Expression Levels in Pathology

Recent analyses of long-range neutrophil migration in 3D chamber devices have provided insights into the striking interplay between the strengths of chemoattractant gradients and the desensitization of chemokine receptors in a GRK-dependent manner [10]. This mechanism appears to control haptotactic migration along surface-bound chemokine gradients but not cell chemotaxis in soluble gradients [10]; in other words, the nature of the chemoattractant and its temporal variations might affect GRK activation and subsequent cell behaviour. Moreover, elevated chemoattractant concentrations were reported to be able to promote cell chemorepulsion or fugetaxis in certain contexts [154]. For instance, increases in the absolute concentration of CXCL8 at sites of inflammation might contribute to avoiding potential tissue damage through neutrophil chemorepulsion [155].

**Figure 3:**
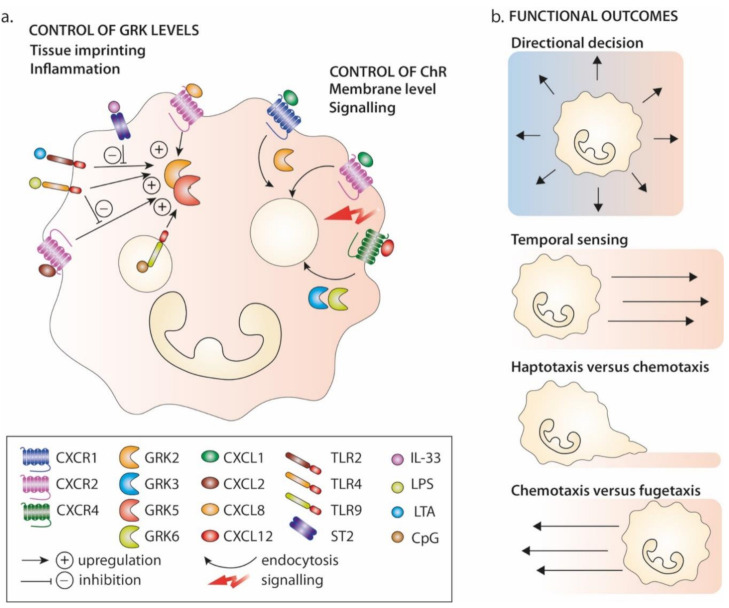
GRK regulation and function in neutrophils. (**a**) Neutrophils are among the immune cells that express GRK2, 3, 5 and 6 at the highest levels. GRK may participate in the tissue-driven imprinting of neutrophils by notably modulating chemokine receptors expression level and signalling. Several exogenous ((LPS via TLR4) [5,158], CpG oligodeoxynucleotide (via TLR9) [159], (LTA via TLR2) [157]) and endogenous (CXCL2 [162] or CXCL8 and LTB4 in the presence of inflammatory chemokines [156]) inflammatory signals upregulate GRK2 and/or GRK5 protein expression levels (depicted by the “+” symbol). LPS [162] and interleukin (IL)-33 (through ST2 receptor engagement) [160] can limit this increase (depicted by the “−” symbol). The cell colour gradient represents the integration of GRK relative levels and activities into chemokine receptor levels and function. (**b**) GRK-dependent regulation contributes to the migratory outcomes of neutrophils from directional migration to temporal sensing (e.g., haptotaxis or chemotaxis, as well as fugetaxis in some contexts). The colour gradients represent the chemokine concentrations, different colours meaning different chemokines.

The spatial and temporal contributions of chemokines and/or other chemoattractants (e.g., C5a, LTB4 and formylated peptides) to the migratory modes of neutrophils either during homeostasis or in the course of the transmigration of neutrophils through the endothelium into inflamed tissues has been recently discussed by Lämmermann and Kastenmüller [136]. Several studies have identified changes in GRK expression levels induced by pathological conditions, of which sepsis, a complex clinical condition that arises in response to severe microbial infection or extensive tissue damage, is the most studied condition. Neutrophils from septic patients have been reported to express increased levels of GRK2 and GRK5, a pattern that was reproduced in vitro upon neutrophil exposure to CXCL8 or LTB4 in the presence of inflammatory cytokines [156] (Figure 3a). In experimental mouse models of sepsis, GRK2 upregulation in neutrophils was associated with the decreased expression of CXCR2 at the cell surface and a subsequent decrease in the migration of neutrophils to infection sites, resulting in shorter survival of the mice [5,157,158,159]. These observations most notably have led to the identification of several cellular receptors as potential susceptibility factors for the pathophysiology of sepsis, such as Toll-like receptors (TLRs). However, interleukin-33 (IL-33) has therapeutic potential in view of its capacity to antagonize the TLR4-dependent modulation of interrelated GRK2 and CXCR2 activities in a mouse model of sepsis and in human neutrophils [160], as also suggested for fibrates, which are ligands of peroxisome proliferator-activated receptor-alpha [161]. An early study suggested an additional level of control as a possible feedback control loop through which the engagement of TLR4 by LPS limited CXCL2-induced GRK2 and GRK5 upregulation and the subsequent CXCR2 desensitization [162]. Recent studies capitalized on unbiased methods to identify biomarkers and susceptibility factors for sepsis [163], as notably illustrated by a comparative kinome profiling approach in patients suffering from systemic inflammatory response syndrome, suggesting that different kinases are activated according to the infectious states of the patients [164], although the small effective sample of patients does not allow us to interlink these changes to the manifestation or progression of the disease.

In other pathological contexts, GRK2 is a very relevant signalling hub, such as in heart failure, where GRK2 protein expression levels are increased at late stages after myocardial infarction [34] but are decreased early after ischaemia. This downmodulation concomitant with PI3K/Akt signalling represents a detrimental event that, when prevented, may attenuate myocardial injury [73]. Although less well-documented than the activity of GRK2, GRK3 and GRK5 and 6 proteins are also regulated by their subcellular location and expression levels, notably heat shock proteins (e.g., Hsp90) and proteasome-dependent pathways, as reviewed elsewhere [165]. In addition, several cell systems have also provided insights into the interplay between GRKs and non-GPCR pathways, as reviewed elsewhere [166], where the binding of GRKs to activated GPCRs promote interactions with intracellular non-GPCR proteins and stimulate GRK-catalyzed phosphorylation, as illustrated by downstream CXCL12/CXCR4 signalling [8]. In addition to this cooperative process, the GRK-dependent phosphorylation of CXCR4 C-ter was shown to be required for the reported TCR-mediated transactivation of CXCR4 in a feedback loop where TCR-activated tyrosine kinases activated GRK [167]. Altogether, these findings suggest that GRKS levels are important determinants of the migration strategies adopted by neutrophils when sensing chemoattractant signals that may participate in their tissue-related phenotypic and functional specialization (Figure 3b).

## 5. Concluding Remarks and Perspectives

The past two decades witnessed conceptual advances in identifying the multifaceted nature of GRKs. However, in addition to the foundational paradigm that GKRs participate in receptor desensitization, studies of their roles as positive regulators of the chemotactic responses of immune cells revealed some conceptually novel leads, including the importance of the phosphorylation barcoding of a receptor in guiding cellular physiological outcomes and modes of migration. These recent findings have raised important questions regarding the diversity of both chemoattractant signals encountered by immune cells and their GPCRs: How do GRKs regulate chemokine/chemokine receptor systems? How do different GRKs operate in concert or sequentially to mediate GPCR functions? How do networks of chemoattractants, receptors and GRKs respond to metabolic or circadian clocks? Information on the mechanisms regulating the GRK levels and activation during homeostasis and inflammation is lacking. In addition, the reported roles of several chemokine receptors in cell proliferation and tissue regeneration have prompted the question of how GRKs are associated with these critical functions. In line with this, studies investigating how atypical chemokine receptors are regulated by GRKs and how their expression, notably by the endothelium, contributes to environmental cues will provide further insights into how this complex network operates. A second important conclusion from these studies is that GRKs appear to be neither promiscuous nor interchangeable with regard to a particular chemokine/chemokine receptor system: all various functional and physiological outcomes might depend on changes in the expression or the activation of a specific GRK. Future studies are required to define whether and how these changes occur, along with the development and activation status of leukocytes in relationship to the tissue environment. In addition, deciphering the mechanisms that fine-tune the expression levels of GRKs in inflamed sites will increase our understanding of how adversely or positively GRKs affects disease development. All of these questions are also particularly important for the development of future therapeutic strategies.

## Figures and Tables

**Table 1:** G protein-coupled receptor kinases (GRKs) and their chemokine receptor partners.

GRK	Chemokine Receptor	Signalling Pathway/Cellular Response
**GRK2**	ACKR3	CXCL12 scavenging [2]
	CCR9	Receptor desensitization [3]
	CXCR1	Receptor desensitization [4]
	CXCR2	Receptor desensitization [5]
	CXCR4	Receptor desensitization [6,7]
		Chemotaxis [8]
**GRK3**	ACKR4	Recruitment of arrestins [9]
	CCR7	Receptor desensitization [10]
		Arrestin signalling [11]
	CXCR4	Arrestin signalling [8]
		Receptor desensitization [6,7,12,13]
		Recruitment of arrestins [14]
**GRK 5**	ACKR3	CXCL12 scavenging [2]
**GRK 6**	CCR7	Receptor desensitization [10,15]
		Arrestin signalling [11]
	CXCR1	Receptor desensitization [4]
	CXCR4	Receptor desensitization [6,7]
		Arrestin signalling [8]

**Table 2:** GRK partners and their functions in immune cells and inflammation.

GRK	Partner (s)	Partner’s Associated Signalling Pathway/Cellular Response
**GRK2**	NF-κB p105 subunit and inhibitor (IκB-α) phosphorylation	TLR4-induced and Tumour Necrosis Factor-α (TNF-α) pathways [20,79,80]
	p38 phosphorylation	P38 mitogen-activated protein kinases (MAPK) pathways [81,82]
	Raf1, MEK1, ERK2, RhoA, RKIP, GIT	Extracellular signal-regulated kinase (ERK) pathways [77]
	Serine-threonine kinase Akt phosphorylation	Akt-Nitric Oxide (NO) pathways [83,84]
	Ezrin/radixin/moesin phosphorylation	Actin cytoskeleton [85,86]
	ADP ribosylation factor (ARF)-specific GTPase-activating proteins (GIT)	Focal adhesion dynamic [78,87]
	Histone deacetylase 6 (HDAC6) phosphorylation	Microtubules network [88]
	Heat shock protein 90 (Hsp90)	Regulation of GRK expression [89]
	Receptor-regulated Smads (R-Smads) phosphorylation	Transforming growth factor beta (TGF-β) pathways [90,91]
**GRK3**	HSP90	Regulation of GRK expression [89]
**GRK5**	ERM (moesin phosphorylation)	Actin cytoskeleton [92]
	GIT1	Regulation of receptor endocytosis [87]
	HSP90, HSP70	Regulation of GRK expression and CXCR4 endocytosis
	NF-κB p105 subunit and IκB-α phosphorylation	TLR4-induced and TNF-α pathways [89,93]
	Src Tyrosine kinase	GRK phosphorylation and neutrophils exocytosis [94]
**GRK6**	HSP90	Regulation of GRK expression [89,95]

## Data Availability

Not applicable.
